# Accelerating Detection of Variants During COVID-19 Surges by Diverse Technological and Public Health Partnerships: A Case Study From Indonesia

**DOI:** 10.3389/fgene.2022.801332

**Published:** 2022-01-28

**Authors:** Ariel Pradipta, Meutia Ayuputeri Kumaheri, Lilik Duwi Wahyudi, Anindya Pradipta Susanto, Harryyanto Ishaq Agasi, Anuraj H. Shankar, Pratiwi Sudarmono

**Affiliations:** ^1^ Genomik Solidaritas Indonesia (GSI) Lab, Jakarta, Indonesia; ^2^ Indonesia Medical Education and Research Institute, Faculty of Medicine Universitas Indonesia, Jakarta, Indonesia; ^3^ Eijkman-Oxford Clinical Research Unit, Jakarta, Centre for Tropical Medicine and Global Health, University of Oxford, Oxford, United Kingdom; ^4^ Indonesian Society for Clinical Microbiology, Tangerang, Indonesia

**Keywords:** SARS-CoV-2, variant, whole-genome-sequencing, GISAID, turn-around-time, genome sequencing platform, Indonesia

## Abstract

Early detection of Severe Acute Respiratory Syndrome Corona Virus 2 (SARS-CoV-2) variants and use of data for public health action requires a coordinated, rapid, and high throughput approach to whole genome sequencing (WGS). Currently, WGS output from many low- and middle-income countries (LMIC) has lagged. By fostering diverse partnerships and multiple sequencing technologies, Indonesia accelerated SARS-CoV-2 WGS uploads to GISAID from 1,210 in April 2021 to 5,791 in August 2021, an increase from 11 submissions per day between January to May, to 43 per day between June to August. Turn-around-time from specimen collection to submission decreased from 77 to 5 days, allowing for timely public health decisions. These changes were enabled by establishment of the National Genomic Surveillance Consortium, coordination between public and private sector laboratories with WGS capability, and diversification of sequencing platform technologies. Here we present how diversification on multiple levels enabled a rapid and significant increase of national WGS performance, with potentially valuable lessons for other LMICs.

## Introduction

The Corona Virus Disease-2019 (COVID-19) pandemic has continued (Wang et al., 2021; World Health Organization, 2021) driven by low vaccination rates, premature reduction in physical distancing, and the emergence of variants. By early 2021, the World Health Organization (WHO) had designated at least four variants of concern (WHO Team Emergency Response, 2021) and spurred global efforts to quickly detect and track SARS-CoV-2 variants by whole genome sequencing (WGS). While high-income countries could meet WGS targets for cases and priority groups (e.g., breakthrough infections), most low- and middle-income countries (LMIC) have lagged. Many attribute this to gaps in financial resources, laboratory equipment, and staff. However, we propose a more foundational cause in low-resource settings tendency toward one-dimensional strategies and technologies. Here we suggest a strategy wherein diversity in WGS technologies may be more successful than single technology solutions, and offer the experience in Indonesia as a case study.

Indonesia is the fourth most populous country in the world and the largest in South-East Asia by population and geography, with unique pandemic challenges across diverse settings. By bringing together a mix of technologies and public health experts from both the public and private sectors, high throughput workflows and technologies were established in a low-resource setting. This catalyzed an acceleration to nearly 4 times more SARS-CoV-2 WGS per day, and a 93% reduction in the delay from specimen collection to sequence availability in the GISAID database. This allowed for swift detection of the dominant variants in Indonesia during a large surge of COVID-19 cases from June 2021 onwards.

### Establishing a National Sequencing Consortium and Challenges

**FIGURE 1:**
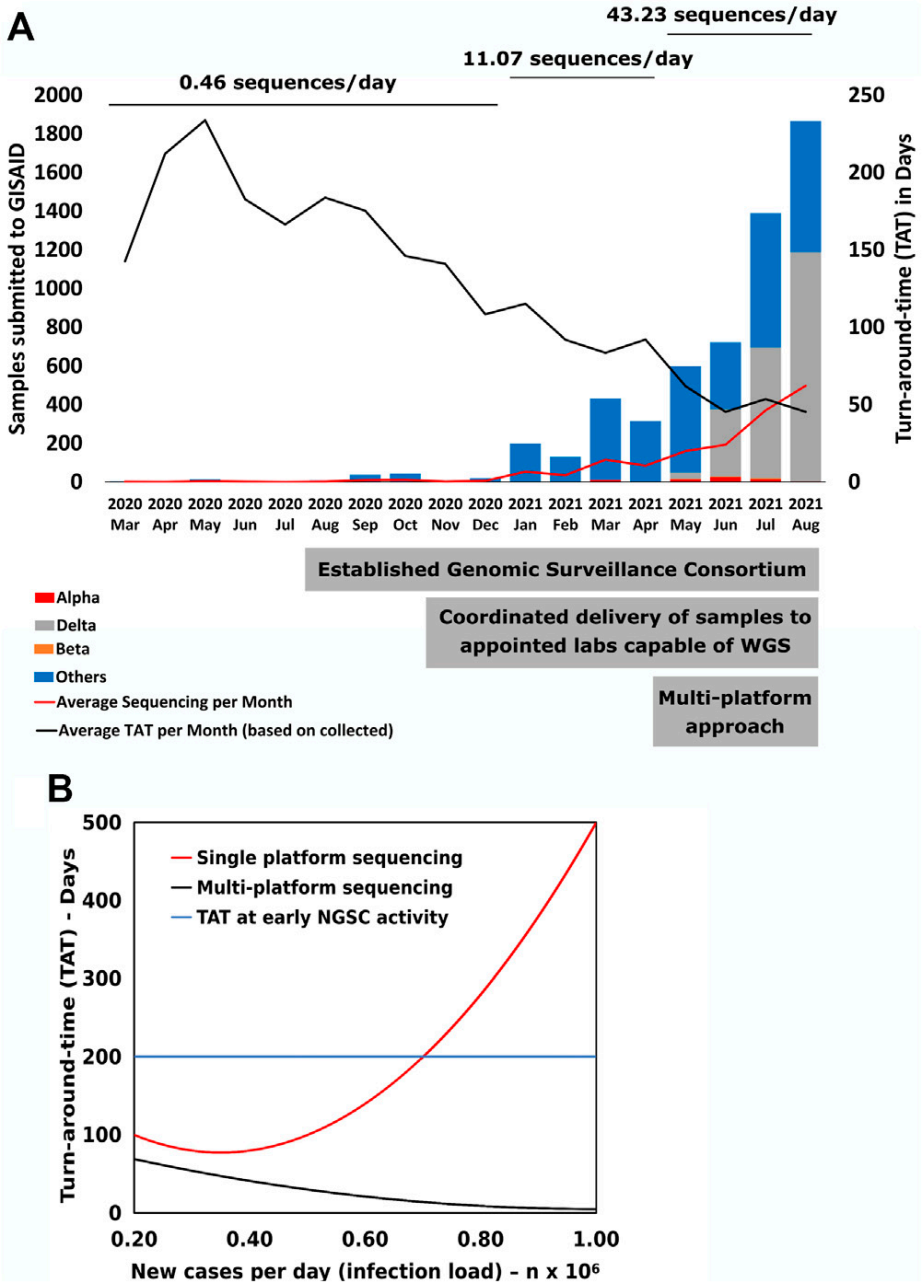
SARS CoV-2 genomic sequencing performance. **(A)** Monthly changes of total number of submitted SARS-CoV-2 sequences to the GISAID database. The average number of sequences per day was calculated by the total number of sequences submitted during that time period divided by the total number of days. The significant steps taken by the country are displayed at the base of figure. **(B)** Mathematical model to calculate changes in Turn-around-time (TAT) of sequencing from specimen collection date to GISAID submission date (*Y*-axis) based on sequencing load associated with the number of new cases (X-axis).

In 2020 the National Genomic Surveillance Consortium (NGSC) was established by the National Institute of Health Research and Development (NIHRD), Ministry of Health (MoH), and Ministry of Research and Technology (MRT). The NGSC created a network comprised of primarily academic partners coordinated by NIHRD, with 13 utilizing Illumina systems for WGS (11 MiSeq, 2 NextSeq). From March 2020 to April 2021, the Consortium had recorded 1,210 SARS-CoV-2 sequences; this was 0.073% of 1,668,360 recorded infections in Indonesia, with a monthly average of 140 days from specimen collection to sequence uploading to GISAID. This proportion of SARS-CoV-2 infections that were sequenced was far below recommendations (European Centre for Disease Prevention and Control, 2021). Fortunately, during that time period, there were few variants of interest or concern (Figure 1A). This inadequate sequencing throughput was due to several challenges such as poor reagent availability, inconsistent library preparation quality, and lack of high throughput streamlined workflows and laboratory information systems. In addition, the lower number of specimens processed and loaded per flow cell led to higher overall costs per sequence, thereby exacerbating already stretched resources. Finally, the general perspective that sequencing technology was primarily for research rather than public health action led to fewer resources committed to WGS efforts.

### Strategic Enhancement of Variance in Sequencing Tools and Public Health Partnerships

To meet the need for rapid WGS, the MoH and NGSC diversified the approach by broadening the network to include a private sector social enterprise doing high-throughput PCR diagnostics, and repurposing its workflows for real-time sequencing. This catalyzed more diversity within the network in five core areas: 1) introduction of the latest Oxford-Nanopore Technology (ONT); 2) increased use of high throughput workflows; 3) more streamlined information management and bioinformatics; 4) transition to sequencing primarily as a public health tool for pandemic mitigation; and 5) increased inter-laboratory cooperation coupled with friendly competitive challenges. Moreover, to adapt to a wide variety of specimen sources, guidelines for standardized sample delivery conditions were created that streamlined specimen preparation and reporting.

### Impact on Viral Sequencing Output and Inference

This additional diversity of tools in combination with the well-communicating network enabled an increase from an average of 11 sequences per day submitted to GISAID from January to May 2021, to 43 per day from June to August (Figure 1A), a 3.9-fold increase. Analysis of time trends using linear spline regression revealed this acceleration was 38% attributable to direct WGS contributions from the new labs using new approaches, and 62% to indirect increased output from other NGSC members associated with items 2) to 5) above. This overall increase was accompanied by a progressively decreasing time from specimen collection to sequence upload, with a sharp improvement following the increase in diversity of approaches.

We note that from the 3,125 specimens collected from May to August 2021, 71% (36 Alpha, 11 Beta, 2189 Delta) were variants of concern, with the Delta variant being dominant, and progressively and rapidly displacing the previously dominant B.1.466.2 variant in Indonesia. Moreover, the Delta variant was present in various sources, notably in children and rural locations. These gains in WGS rate and actionable knowledge underscore how diversification in laboratory and public health ecosystems can rapidly have broad impacts. The results translated to rapid and effective communication to authorities for COVID-19 testing and tracing efforts.

### Role for Complementarity of Sequencing Platforms

We highlight that from June to August 2021, NGSC laboratories using ONT were able to submit 870 (37.8%) WGS results out of the national total of 2,301. Moreover, during this same period the average period from specimen collection to GISAID sequence upload, the turnaround time (TAT), was 8.6 days for high throughput setups. In comparison, the TAT for other laboratories was 36.5 days, although improving rapidly. We note that previous studies have shown the ability of ONT to provide sequencing data in 24 h with workflows of 8 h, allowing for same-day WGS. Other advantages include lower cost of entry for equipment, smaller batch sizes with fewer cost implications, reusable flow cells (Lipworth et al., 2020), and real-time monitoring (Bharagava et al., 2019) of sequencing reads to detect and react to suboptimal specimen processing. The ONT sequencing community provides quick access to specific workflow assistance for base-calling software (Guppy), and neural-network-based software for sequence assembly (Medaka), which are also optimized with a bioinformatics pipeline for subsequent dry analysis. Lineages can thereafter be assigned algorithmically using software such as Pangolin, which implements the Pango nomenclature system (Makałowski and Shabardina, 2020). Despite heterogeneity in specimens from diverse sources such as health centers, walk-in patients, quarantine centers, and pediatrics surveillance, most samples with ONT produced sequence reads with an average coverage of 83.75% with an average read depth of 337x, sufficient for 98% to be given a proper PANGO-based lineage for variant classification.

Other platforms using sequence-by-synthesis approaches such as Illumina or MGI (Kim et al., 2021) also offer advantages, including a wider installed base of machines, higher capacities per flow cell, and more third-party reagent options, along with excellent support. Still, other systems provide advantages for longer reads, such as Pacific Biosciences (Quail et al., 2012). To optimize speed, reliability, and resilience in serving WGS needs during the pandemic, an ecosystem with a multi-platform approach may prove advantageous compared to a single approach.

The challenges to transform WGS into a public health tool were highlighted during a surge of new cases in Indonesia that required high daily throughput and lower TAT. The increased demand combined with increased number of cases inversely affected WGS output due to capacity saturation. To better understand these dynamics, we used the results (Figure 1B) to model how increased load influenced the TAT. As cases increase to over 7,000 per day, the model showed that the single platform lowest TAT would be 77 days, too long to inform appropriate public health action. However, by introducing a multi-platform approach, the TAT could be reduced to 5 days. Overall, increased surveillance efforts will be required to properly and rapidly identify populations vulnerable to variants, for example, those with decreased immune function or high exposure, such as those older than 65 years, health workers, or other yet undefined attributes at the individual and population level. As an RNA virus, SARS-CoV-2 maintains high mutation rates and, combined with unique selective pressures in each infected individual and community, can quickly give rise to novel viral populations (Korber et al., 2020; Okada et al., 2020). Efforts are ongoing to gather more accurate reports on vaccination status, comorbidity, clinical outcome, and other relevant information to uncover the possible impacts of variant-related COVID-19 surges (Ramasamy et al., 2020; Challen et al., 2021; Davies et al., 2021; Ozono et al., 2021).

## Conclusion

The need for genomic sequencing capability dramatically increased due to the COVID-19 pandemic. In the middle of 2020, SARS-CoV-2 variants presented a significant challenge to pandemic management. Thus, the ability to detect new variants and their spread was crucial for proper public health responses. Indonesia managed to submit SARS-CoV-2 genomic sequences to GISAID in early 2021. However, the speed and amount of genomic sequencing in Indonesia were not adequate to meet the suggested minimum requirement for detection of new variants. The need for proper genomic sequencing was further highlighted by the growing number of variants of concern in early 2021.

We report that Indonesia successfully improved the total number of SARS-CoV-2 sequences submitted to GISAID and rapidly reduced the TAT. This success could be credited to an enhanced ecosystem of laboratory technology and public health partnerships. Moving forward, we recommend several additional improvements. First, substantial communication is required to ensure specimen preparation and delivery standards. Second, for scaling-up capacity, it is clear that preference would be for more mobile sequencing platforms that could reach different parts of the Indonesian archipelago, and isolated regions as seen in many LMICs. Third, there is a need for agile policy wherein requirements for minimum sequencing and meta-data and reporting can be adjusted to better detect emergent variants. In Box 1, we list our conclusions and recommendations based on our case study in Indonesia.

Transition to the Delta variant was rapid, occurring within just a few weeks in the Asian megacity of Jakarta. We suggest the recommended actions are crucial for early detection of variants, such as Omicron, and enable timely decisions to mitigate the potential impact.

Box 1 **Recommendations to accelerate SARS-CoV-2 whole genome sequencing in low- and middle-income countries.**

• Rapidly establish a SARS-CoV-2 sequencing network.
• Deploy multiple sequencing platforms.
• Establish high throughput workflows and laboratory information systems.
• Build public health partnerships including private and public sector entities.
• Focus on coordination between interdisciplinary teams.

## Data Availability

The original contributions presented in the study are included in the article/Supplementary Material, further inquiries can be directed to the corresponding author.
